# Linking Innovative Human Capital, Economic Growth, and CO_2_ Emissions: An Empirical Study Based on Chinese Provincial Panel Data

**DOI:** 10.3390/ijerph18168503

**Published:** 2021-08-11

**Authors:** Xi Lin, Yongle Zhao, Mahmood Ahmad, Zahoor Ahmed, Husam Rjoub, Tomiwa Sunday Adebayo

**Affiliations:** 1 Business School, Hohai University, Nanjing 211100, China; zhao_yongle@163.com; 2 Business School, Guilin University of Technology, Guilin 541004, China; 3 Business School, Shandong University of Technology, Zibo 255000, China; mahmood@sdut.edu.cn; 4 Department of Economics, Faculty of Economics and Administrative Sciences, Cyprus International University, Mersin 10, Haspolat 99040, Turkey; zahoorahmed83@yahoo.com; 5 Department of Accounting and Finance, Faculty of Economics and Administrative Sciences, Cyprus International University, Mersin 10, Haspolat 99040, Turkey; hrjoub@ciu.edu.tr; 6 Department of Business Administration, Faculty of Economics and Administrative Science, Cyprus International University, Nicosia, Northern Cyprus, Mersin TR-10, Turkey

**Keywords:** innovative human capital, CO_2_ emission, Chinese provinces, economic growth

## Abstract

To study the economic and environmental effects of human capital, previous studies measure human capital based on education; however, this approach has many shortcomings because not all educated people are innovative human capital. Hence, this study introduces the concept of innovative human capital by developing a new index that measures human capital based on the number of patents every one million R&D staff full-time equivalent. After this, this paper studies the impact of innovative human capital on CO_2_ emissions in China. The provincial panel data of 30 Chinese provinces from 2003 to 2017 is analyzed using the fixed effect, ordinary least squares, and the system generalized method of moments (SYS-GMM). The analysis revealed that innovative human capital alleviates environmental deterioration in China. The findings unfold the existence of the environmental Kuznets curve (EKC) considering innovative human capital in the model. It implies that Chinese economic development will eventually support environmental sustainability if China continues to develop its innovative human capital. Among the control variables, economic structure, population density, and energy intensity stimulate environmental degradation by increasing CO_2_ emissions. However, FDI has a negative relationship with CO_2_ emissions. Lastly, the study proposes comprehensive policies to increase innovative human capital for environmental sustainability.

## 1. Introduction

Economic growth is humans’ eternal objective and the focus of economists across the world. However, with the development of a global economy since the 1960s, environmental problems have become serious and concerned various international organizations and countries. Thus, in 1980, the United Nations Environment Program (UNEP) appealed for sustainable development with two other global organizations. The Earth Summit was held in Rio and published the very famous declaration “Rio Declaration on Environment and Development” in 1992. Afterward, the Kyoto protocol and Paris agreement were signed under the United Nations (UN) framework convention on climate change [1]. China actively joined the two agreements and has made efforts to pursue green growth since then. The idea of green growth has originated from the Asia and Pacific Region. Green growth provides the idea of harmonizing growth and ecological sustainability while upgrading the eco-efficiency of growth and increasing the synergies between the economy and the environment [2]. At present, the Chinese economy is transferring to a higher-quality, more balanced, and greener growth path. Chinese central government has taken many measures to reduce the environmental pressures caused by economic growth. China has made some progress in the fields, such as energy and carbon intensities, per capita carbon emission intensities and environmentally related taxes, etc. However, there are lots of problems waiting to be solved in the largest CO_2_ emitting country.

**Figure 1:**
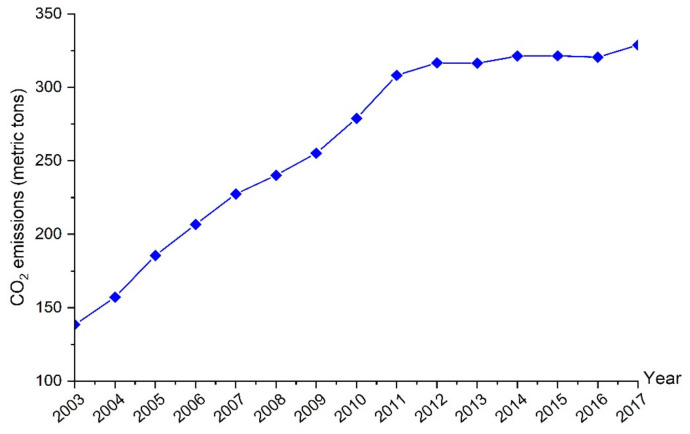
Annual trend of CO_2_ emissions in China over 2003–2017. Data source: China energy statistical yearbook (https://data.cnki.net/Yearbook/ accessed on 16 June 2021).

According to the 2011 OECD Ministerial Council Meeting, innovation sustained by a sturdy intellectual property rights system supports economic growth, conserves the environment, and creates employment. In this regard, China has invested so much in scientific and technological innovation for decades and ranked second for global gross expenditure on R&D (GERD). According to the statistics, the environment-related patents have increased 15 times for the period 2000–2012 in the context of China. This increase is much more than the increase in the innovation of the OECD countries. However, the inventions’ quality and protection still need to be improved. In addition, the share of GERD in GDP and the share of research personnel in the overall employment are also less than many OECD nations [3]. China is the number one country in terms of total CO_2_ emissions in the world; meanwhile, per capita carbon emission has also kept increasing [4]. As clearly shown in Figure 1, total carbon emissions increased from 138.47 metric tons to 328.86 metric tons over the period of 2003–2017 in China. In 2007, the Intergovernmental Panel on Climate Change (IPCC) stated that the CO_2_ emissions emitted by humankind contribute to 90% of global warming. The current technology is unlikely to decrease carbon emission compared to other pollutants [5]. It is noteworthy that more effective technologies still depend on relentless innovation by Research and Development (R&D) personnel. R&D personnel are defined as “specific human capital whose knowledge and skills are less transferable and have a narrower scope of applicability” [6]. This specific human capital can also be called as innovative human capital, and it is a unique resource to promote productivity and technological progress. Thus, innovative human capital plays a vital role in economic development [7]. More precisely, innovative human capital can be defined as a kind of heterogeneous capital that always possesses cutting-edge knowledge and skills in a specific professional field, continuously carries out innovative activities, and obtains innovative output, so that the marginal income can continue to increase.

Although innovative human capital has the strongest innovative ability to create clean production technologies compared to the other kinds of human capital, few scholars paid attention to the relationship between innovative human capital, green growth, and environmental sustainability. At present, many researchers are still mainly discussing the relationship between the quantity of human capital, economic growth, and environmental quality. They usually use educational attainment to measure the quantity of human capital and neglect the quality difference of human capital. However, the quality of human capital may be more relevant to environmental sustainability and green growth. The quality aspects of human capital may have greater potential in explaining growth [8].

The measurement of human capital, including innovative human capital has always been a controversial issue in academic circles. In the past, many scholars used people with tertiary education to represent innovative human capital [9,10,11,12]. Although this measuring method is widely used, it may cause the following several problems: first, this method omits the qualitative difference among the people with tertiary education because the people’s educational attainment only reflects the differences in the amount of human capital [13]. Second, college students are usually included in the number of people receiving higher education, which amplifies the stock of innovative human capital to a large extent. Third, not all the people who have received higher education are innovative human capital, and those who are engaged in scientific and technological work are the main part of innovative human capital. Thus, it is very necessary and meaningful to compute the innovative human capital by addressing all these weaknesses and probe the influence of innovative human capital on CO_2_ emissions in China. It is a vital point for China to strictly control CO_2_ emissions and effectively execute green development. To avoid the above problems, this paper followed the footsteps of Romer [14] and mainly considered science and technology staff when measuring the innovative human capital.

Hence, this new proxy for innovative human capital will more likely capture the ability of innovative human capital to promote innovation which can play a mounting role in increasing energy efficiency and output, decreasing environmental degradation. In addition, this will reduce the chances of unreliable results and misleading policies that may arise due to the use of inappropriate measures of human capital. For example, using the education-based human capital, the recent work of Ahmed et al. [15] found that human capital is positively linked with emissions in Latin American countries. We believe that such surprising outcomes can arise due to the weaknesses of previous proxies of human capital. Additionally, education-based measures are mainly based on the view that education promotes awareness that leads to environmental sustainability; however, our measure directly promotes innovation and technological advancement, which are more relevant for environmental sustainability.

Against this backdrop, the authors intend to explore the relationship between innovative human capital and CO_2_ emissions in China using an appropriate measure of innovative human capital. The article uses 15-year Chinese provincial panel data to examine the impact of innovative human capital on emissions in an EKC framework. The study extends the literature and measures innovative human capital by developing an index based on the number of patents every one million R&D staff full-time equivalent. The study has both theoretical meaning and practical applications. Concerning theoretical contribution, the paper will enrich and strengthen the present literature on human capital and environment nexus. It is conducive for us to better understand the impact mechanism of human capital for green growth. On the practical meaning, the research is aiming at providing some advice for the Chinese government to formulate more targeted strategies towards environmental sustainability and green growth.

Additionally, this work is equally important for other developed and developing countries because it presents and discusses a new measure of innovative human capital. In recent literature, studies linking innovative human capital with different economic and environmental indicators are growing both in China and other countries. Thus, the methodology used in the study can be followed for measuring innovative human capital in other countries and regions. This will be helpful to apprehend the role of innovative human capital in economic development and environmental sustainability and formulate appropriate policies to achieve environmental and economic objectives.

The remainder of this article is arranged as follows: Part 2 discusses theoretical background and literature review. Part 3 presents the model and variables. Part 4 presents the empirical strategy. Part 5 is about the interpretation and discussion of results, and the last part (Part 6) presents the conclusion and policy directions.

## 2. Literature Review and Hypothesis Development

### 2.1. Innovative Human Capital and CO_2_ Emissions Nexus

Analyzing the impact of human capital on environmental quality has recently gained some attention from scholars. An increasing body of literature has focused on explaining the environmental impact of human capital by using the traditional education-based proxy [12,16,17]. Scholars have explored the relationship between human capital and environmental issues using both the panel and the single country-level data. From a panel perspective, Yao et al. [18,19] used the data of 20 OECD economies and found that educated people prefer clean energy consumption over dirty energy. They uncover that advanced human capital measured by tertiary education has a negative influence on CO_2_ emissions i.e., an extra year of tertiary schooling is connected with a reduction in emissions between 50.1% and 65.8%.

Alvarado et al. [16] also found that economic development cannot reduce energy consumption from fossil sources, but human capital does decrease the non-renewable energy in 27 OECD countries from 1980 to 2015. Pablo-Romero and Sánchez-Braza [20] empirically studied the relationships between energy, physical, and human capital in a larger region including OECD, NAFTA, BRIC, East European, East Asian, and EU15 countries. They found that there are substitutability relationships between human capital and energy utilization. Khan [21] argued that the influence of economic development on emissions is dependent upon the human capital level, after a certain level of human capital, CO_2_ emissions will be reduced, and environmental awareness and friendly technologies will be promoted. Hao et al. [22] specially investigated the effects of human capital on CO_2_ emissions for G7 countries for the period 1991–2017 and discovered that human capital can reduce CO_2_ emissions. Khan et al. [23] also concluded that human capital can enhance renewable energy consumption of G7 countries by analyzing the data from 1995 to 2017. In addition, Khan et al. [24] found that improvement in human capital can intensify the negative relationship between CO_2_ emissions and fiscal decentralization in G7 countries. The positive role of human capital in promoting renewable energy consumption was also found in some studies for African countries [25,26].

Proceeding to the research on individual countries, Mahmood et al. [27] used the EKC model to empirically explore the effects of economic growth and renewable energy on CO_2_ emissions by adding human capital for Pakistan and discovered that the human capital mitigates CO_2_ emissions. Bano et al. [28] explored the human capital and emissions nexus in Pakistan and revealed that the human capital alleviates emissions without hindering economic growth. Ahmed et al. [4] investigated the effect of natural resources, human capital, and urbanization on environmental quality in China and found that human capital mitigates environmental deterioration and has a moderating effect in promoting sustainable urbanization. Xin and Lyu [29] proved that the EKC model can explain the nexus between technological innovation and pollution in China’s major cities, and human capital strengthens the effect of technological innovation on pollution. Wu and Tian [30] also confirmed the EKC hypothesis at the provincial level in China and pointed out that intermediate-level education is positively correlated with environmental pollution, but higher-level education has the opposite impact. However, the finding of Huang et al. [31] is partly different from Wu and Tian [30]. They separated human capital into four categories, including primary, knowledgeable, skilled, and institutional, and discovered that these four types of human capital have negative impacts on carbon emissions intensity among the eastern, central, and western regions in China [29]. Table 1 presents the summary of literature on human capital and environmental degradation.

Synthesizing the above shreds of evidence, we hypothesize that innovative human capital can promote environmental sustainability through relevant research and development activities. Hence, we formulate the following hypothesis:

**H1.**  *Innovative human capital poses a positive impact on environmental quality.*

### 2.2. Economic Growth and CO_2_ Emissions Nexus

The relationship between economic growth and environmental degradation has been a hot issue over the last two decades. In 1991, Grossman and Krueger [36] first used the concept of the EKC to study the impact of economic growth on environmental quality and found an inverted U-shaped relationship between per capita GDP and pollution. Since then, a bulk of studies devoted to scrutinizing the association between GDP and environmental quality based on EKC, but conclusions are different. For instance, Narayan and Narayan [37] investigated the impact of income on environmental quality in 43 countries. Their results confirmed the existence of the EKC as environmental degradation decreases with the increase in income over time. Similar findings are also reported by numerous scholars, for instance, Pata [38] for Turkey, Song et al. [39] for the United States and China, Danish et al. [40] for BRICS countries, Narayan et al. [41] for the panel of 181 countries. Yang et al. [42] also revealed the presence of EKC in OECD countries, suggesting that economic growth causes environmental degradation at an early level of development but improves environmental quality after reaching a certain level.

On the contrary, some researchers believed that the EKC is invalid. For instance, Akbostanci et al. [43] tested the EKC hypothesis using the data of Turkey over 1992–2001. Their results do not support the EKC for both panel and times series data. Likewise, using the spatial econometric approach, Wang and Ye [44] investigated the impact of income on environmental quality in China. Their results unveiled that income monotonously increases CO_2_ emissions, indicating that environmental degradation will not decline with the increase in income. In addition, some studies did not fully unfold the EKC for their entire sample. Al-Mulali et al. [45] found that EKC is valid for upper-middle and high-income countries but not in low and lower-middle income countries. Similarly, Guangyue and Deyong [46] tested the EKC for China’s regional carbon emissions. Their results indicated that EKC is valid for the central and eastern regions but not for the western region. Therefore, we formulate the following hypothesis:

**H2.**  *There is an inverted U-shaped relationship between economic growth and CO_2_ emissions.*

This detailed literature review represents that several studies evaluated the human capital and CO_2_ emissions nexus, but all these studies used the human capital based on education. We have already pointed out the limitations of such a measurement in Section 1. Hence, this study uses a better measurement of human capital and examines its effects on CO_2_ emissions using the EKC framework.

## 3. Theoretical Background and Model Construction

### 3.1. Theoretical Background

The concept of innovative human capital originated from some scholars who thought about the heterogeneity of human capital. They proposed that human capital can be divided into two categories in the form of marginal return: if the kind of human capital increases marginal return during some special period, it will be known as idiosyncratic human capital; conversely, it will be known as coessential human capital [47,48,49]. Based on this opinion, Yao [50] first raised the concept of innovative human capital that he thought can move the production boundary outward. Why does innovative human capital own such an ability to increase production efficiency? The reason is that innovative human capital can innovate knowledge, technology, and information [51]. Subsequently, some scholars took further research on innovative human capital and classified it into many different kinds [52,53]. From the theory of marginal return, the connotation of innovative human capital and idiosyncratic human capital are the same.

Innovative human capital can make an impact on the economy from micro, industry, and also on macro levels. At the micro level, the enterprise system can affect employees’ innovative behaviors from the three aspects, including incentive mechanism, transaction costs, and uncertainty of innovation [54]. More importantly, innovative human capital is the critical determinant of firm performance differential [55]. Enterprises’ competitive advantage is significantly and positively influenced by innovative human capital [56]. Additionally, from the viewpoint of innovation propensity, for small firms, innovative human capital can be more desirable and valuable [57]. University graduates are usually regarded as innovative human capital by researchers around the world. Xia Pan et al. [58] used the number of university graduates to measure higher education and found the total number of university graduates reduce innovation at the provincial level, but the graduates from the reputed higher education institutions increase firm-level innovation at the provincial level in China. Peng [59] undertook deeper empirical research in which he discovered that graduates with master’s degrees can bring radical innovation, and graduates with bachelor’s degrees can push progressive innovation for firms.

At the industry level, Xia Pan et al. [58] further pointed out that elite university graduates are positively connected with the innovation of privately owned enterprises and insignificantly connected with the innovation of the state- and foreign-owned enterprises. However, in high-tech industries, this positive connection was more prominent. Zhu and Li [60] concretely conducted an empirical study of the technological innovative human capital’s impact on the manufacturing industry competitiveness based on the data of Guangdong province in China and found that the technological innovative human capital plays a key role in the innovation of the industry. Besides, some scholars researched the impact of innovative human capital on different Chinese industries like the pharmaceutical manufacturing, e-commerce, and high-tech industries, and they all found that the innovative human capital has a positive impact on these industries to gain competitive advantage [61,62,63].

At the macro level, there is a new trend that innovative human capital (IHC) measured by the number of university graduates has gradually increased the contribution, and the other kinds of human capital have gradually decreased the contribution to Chinese economic growth [9]. The effect of IHC on China’s economic growth is several times more than that of basic human capital [64]. Innovative human capital plays a more and more decisive role in production [65]. Although the Chinese economy has been mainly facilitated by investment, economic growth is increasingly dependent on innovative human capital [66]. Innovative human capital mainly promotes technological progress through technological innovation and is essential to realize sustained and rapid economic development [67]. However, from the perspective of regional comparison, China’s innovative human capital has obvious regional differences [10]. With the continuous deterioration of the environment, it is obviously not enough to focus only on the impact of IHC on economic growth, but it is also essential to explore the role of innovative human capital in environmental protection.

Theoretically, economic growth can affect carbon emissions through three main channels, i.e., scale, composition, and technique [68]. The scale effect implies that more production requires extra material during the early stage of development, which generates more waste and pollution. The composition effect indicates that the level of pollution and raw material pattern used in the manufacturing process depends on the country’s economic sectors. For instance, there will be less pollution in the countries possessing a larger services sector. Therefore, structural transformation along with economic development affects environmental quality. The third channel is the technique effect which implies that more advanced and green technologies create less material-intensive goods and less pollution at a high level of economic growth [69].

### 3.2. Model Construction

The EKC framework proposed by Grossman and Krueger [68,70] has been widely used by scholars in China and other countries and has been verified to varying degrees. Hence, following previous studies [33,71], this paper decides to build the following econometric model based on the EKC framework.

(1) $$y\;=\;\alpha_{0}\;+\;\beta_{1}x\;+\;\beta_{2}x^{2}\;+\;\mu$$

In Equation (1), *y* measures environmental impact, *x* refers to GDP per capita; *α_0_* denotes the constant, $\beta_{1}$ and $\beta_{2}$ are the parameters to be estimated relating to *y*; finally, *μ* denotes the interference term. As the study uses China’s provincial-level panel data and meanwhile considers the factor of innovative human capital influencing CO_2_ emissions, the Equation (1) can be transformed into the following form:

(2) $${CO}_{2_{it}}\;=\;\alpha_{0}\;+\;\alpha_{1}GDP_{it}\;+\;\alpha_{2}GDP_{it}^{2}\;+\;\beta_{1}IHC\;+\;\mu_{it}$$

In Equation (2), subscripts *i* and *t*, respectively, denote province and year. GDP denotes GDP per capita. IHC means innovative human capital and *β_1_* is its parameter.

For alleviating omitted variable bias, we sequentially added several control variables, which are possibly connected with variation in CO_2_ emissions. Therefore, Equation (2) can be represented as follows:

(3) $${CO}_{2_{it}}\;=\;\alpha_{0}\;+\;\alpha_{1}GDP_{it}\;+\;\alpha_{2}GDP_{it}{}^{2}\;+\;\beta_{1}IHC_{it}\;+\;\sum_{j\;=\;1}^{n}\gamma_{j}M_{jit}\;+\;\mu_{it}$$

In Equation (3), subscripts *i* and *t*, respectively, denote province and year. *M* refers to the control variables. In the selection of control variables, after referring to the existing literature, we decided to control four variables, including population density, economic structure, energy intensity, and FDI. Therefore, Equation (3) can be transformed as follows:

(4) $$\begin{array}{l} {CO}_{2_{it}}\;=\;\sum_{j\;=\;1}^{p}\alpha_{j}CO2_{i,t-j}\;+\;\beta_{1}GDP_{it}\;+\;\beta_{2}GDP_{it}^{2}\;+\;\beta_{3}IHC_{it}\;+\;\beta_{4}POP_{it}\;+\;\beta_{5}STRU_{it}\;+\;\beta_{6}ENER_{it}\\ \;+\;\beta_{7}FDI_{it}\;+\;\nu_{i}\;+\;\varepsilon_{it} \end{array}$$

### 3.3. Variables and Data Sources

In the above equation, CO_2_ is the dependent variable. The independent variables are GDP and IHC. Besides, there are four control variables, including POP, STRU, ENER, and FDI. Table 2 provides more detail on these variables.

In Table 1, the data on three independent variables including real GDP per capita, population density, and energy intensity are directly extracted from both Chinese provincial statistical yearbooks and China environmental statistical Yearbooks. The data on the other three independent variables (IHC, economic structure, and FDI) is calculated based on the relative data originating from both Chinese provincial statistical yearbooks and China science and technology statistical yearbooks. The paper’s study period is from 2003 to 2017, so we use the price of 2003 to denote real GDP per capita. The starting period of the research (2003) is selected based on CO_2_ emissions data (some fossil fuel emissions data is unavailable before 2003) and 2017 is linked with the data availability of innovative human capital. For calculating the quantity of CO_2_ emissions, the paper refers to method 1 of IPCC [72] and takes the following formula:

(5) $$CE_{it}\;=\;\sum_{n\;=\;1}^{n}EF_{n}Activity_{itn}$$

In Equation (5), *CE_it_* indicates the CO_2_ emissions of the *i*-th province in the T-year. *EF_n_* denotes the emission factor of the *n*-th source. *Activity_itn_* means the consumption of the *n*-th source of the *i*-th province in the T-year. For all provinces in China, the main fossil energy consumption is oil consumption (including gasoline, kerosene, diesel, and fuel oil), coal consumption (raw coal and coke), natural gas consumption, and cement production. Therefore, the paper will calculate the total annual CO_2_ emissions of the above eight emission sources for 30 provinces (except Tibet) during the period under analysis. The data of the total consumption of the sources can be collected from China energy statistical yearbooks. About the emission factors of different energy sources, the paper refers to “the guidelines for the compilation of provincial greenhouse gas inventories” published by the Chinese national development and reform commission in 2011. The emission factors of the eight energies are shown in Table 3.

Keeping in view the limitations of education-based proxies of human capital discussed in Section 1, this paper follows Romer [14] and measures human capital based on science and technology staff. In all kinds of Chinese yearbooks, there is no complete data of science and technology or R&D staff from 2003 to 2017, but the complete data of R&D staff full-time equivalent were provided for the same period. Therefore, the paper designs an index named “innovative efficiency” to denote the innovative human capital. The index’s formula (developed by the author) is represented as follows:

(6) $${\mathrm{Innovative}\;\mathrm{efficiency}}_{\mathrm{it}}\;=\;\frac{{\mathrm{Total}\;\mathrm{number}\;\mathrm{of}\;\mathrm{patent}}_{\mathrm{it}}}{{\mathrm{Total}\;\mathrm{number}\;\mathrm{of}\;R\&D\;\mathrm{staff}’s\;\mathrm{full}-\mathrm{time}\;\mathrm{equivalent}}_{\mathrm{it}}}\times 10000$$

In this Equation (6), the innovative efficiency represents innovative human capital. The total number of patents_it_ means the amount of the authorized patents of the *i*-th province in the *t*-th year. The total number of R&D staff full-time equivalent*_it_* indicates the amount of the total working hours divided by average annual working hours in full-time R&D jobs of the *i*-th province in the *t*-th year. The data for calculating innovative efficiency was extracted from both Chinese provincial statistical yearbooks and China science and technology statistical yearbooks. Thus, all variables used in this article and their data sources were discussed.

## 4. Econometric Strategy

This study relied on panel data estimation techniques to empirically analyze the impact of economic growth, innovative human capital, population density, economic structure, energy intensity, and foreign direct investment on carbon emissions. Ahmed et al. [15] suggested that panel data estimation models have several advantages over time series data, such as it provides robust results and counters the issue of heterogeneity, multicollinearity, and endogeneity. Thus, following the studies of Al-Mulali et al. [45] and Dogan et al. [73], we employed panel ordinary least squares estimator (POLS) and fixed-effect method for the baseline model. Fixed effect regression accounts for unobserved time-invariant among individual characteristics, and that may lead to biased results.

Further, this study employs the system generalized method of moment (SYS-GMM) developed by Blundell and Bond [74]. The panel ordinary least square and fixed effect model are criticized for the inefficient results due to unobserved correlation with the lags of regressors [75]. To overcome this issue, Arellano and Bond [76] proposed a GMM method to estimate a dynamic panel model that eliminates the countries’ specific heterogeneity by using the first difference of dependent variable. However, in the simulation studies, Blundell and Bond [74] demonstrated that the first difference GMM has poor precision; when the autoregressive parameter is relatively large, and the time-series observations are small, it may lead to large finite sample bias. To counter this issue, Blundell and Bond [74] developed the system GMM, which relies on the lagged difference of a response variable as an instrument for the equations at level and lagged as instruments for the dependent variable equation at the first difference. Following studies of Muhammad [77] and Ibrahim and Ajide [78], a standard SYS-GMM estimator can be stated as follows:

(7) $$\begin{array}{l} {CO}_{2_{it}}\;=\;\alpha \;+\;\beta_{1}(CO2_{it-1}-CO2_{it-2\varphi })\;+\;\beta_{2}(GDP_{it}-GDP_{it-2\varphi })\;+\;\beta_{3}(GDP_{it}^{2}-GDP_{{}_{it-2\varphi }}^{2})\\ \;\;\;\;\;\;\;\;\;\;\;\;\;\;+\;\beta_{4}(IHC_{it}-IHC_{it-2\varphi })+\beta_{5}(POP_{it}-POP_{it-2\varphi })\;+\;\beta_{6}(STRU_{it}-STRU_{it-2\varphi })\\ \;\;\;\;\;\;\;\;\;\;\;\;\;\;+\;\beta_{7}(ENER_{it}-ENER_{it-2\varphi })\;+\;\beta_{8}(FDI_{it}-FDI_{it-2\varphi })\;+\;\eta_{t}\;+\;\mu_{i}\;+\;\varepsilon_{it} \end{array}$$

where 𝑖 exhibits 30 cross-sectional provinces of China, 𝑡 is the time from 2003 to 2017. φ represents auto-regression coefficient which is based on a year lag assumed sufficient to control for past information, *η_t_* and *μ_i_* are time-specific and country-specific effects, respectively, while *ε* denotes error term.

## 5. Results and Discussion

Descriptive statistics given in Table 4 show those carbon emissions in Chinses provinces are less volatile than economic growth. Economic growth increased from 3603 (per capita constant 2003) to 99783 during 2003–2017. The innovative human capital has a minimum value of 264.25 and a maximum value of 6776.56, indicating a significant upward trend. On average, the population density is 436.75, with a maximum value of 3826.0. Additionally, structural changes in Chinese provinces are varying between 11.84 and 59.240. Moreover, the average foreign direct investment is 3%, and it shows a deviation value of 2.338.

Table 5 shows the outcome of the pairwise correlation matrix. It reveals a positive correlation between GDP and CO_2_. Innovative human capital and population density also show a positive correlation with emissions. The correlation between energy intensity and carbon emissions is negative. On the contrary, structural change and foreign direct investment also depict a positive correlation towards carbon emissions.

Table 6 represents the outcome of panel OLS and fixed effects results. The findings specify that economic growth poses a positive effect on CO_2_ emissions in China. The coefficient is significant in both models, indicating that a 1% increase in GDP will increase CO_2_ emissions by 2.479% and 2.376%. Meanwhile, the coefficient of GDP square is negative, which implies that the EKC hypothesis exists in China’s provinces. These findings corroborate with the results of Ahmad et al. [79] for emerging countries, Pata [38] for Turkey, and Ahmed et al. [80] for Japan. Thus, our result supports hypothesis 2 (H2).

Interestingly, the coefficient of innovative human capital is significant and negative at a 1% level, signifying a mitigating effect of IHC on environmental degradation. The result portrays that innovative human capital in China is actively working for the betterment of environmental quality. Thus, our results support hypothesis 1 (H1). This finding contradicts the conclusion of Ahmed et al. [15] who disclosed that education-based human capital negatively affects the environmental quality in Latin American nations because educational attainment in these countries leads to more economic activities and more energy consumption. This also opposes the outcomes of Balaguer and Cantavella [81] who concluded that human capital in Australia increased CO_2_ emissions for the majority of the years from 1950 to 2014. This result also contradicts the claim of Hassan et al. [71] that human capital in Pakistan does not influence environmental quality. Conversely, our finding is in line with some of the studies that illustrated a positive association between environmental quality and education-based human capital, for instance, Ahmed and Wang [33] for India, Ahmed et al. [34] for G7 nations, and Zafar et al. [32] for the United States.

Our outcome is unique and different from previous studies since instead of using the traditional education-based human capital which decreases or increases environmental degradation in previous studies, we employed innovative human capital based on R&D staff which is more relevant because it is directly linked with innovation. Thus, innovative human capital can improve environmental quality in different ways, such as increasing environmental-related technological innovation and sustainable usage of natural resources [69]. Therefore, innovative human capital can be used as a valuable tool to cope with environmental challenges. Previous studies on human capital and environment nexus illustrate different findings possibly because human capital in those studies is based on education and covers the quantity dimensions rather than quality dimensions. Education and associated awareness may pose a very small influence on environmental quality compared to the innovation and technological advancement that are traits of our innovative human capital. Indeed, innovation and technological advancement can influence technological efficiency and environmental quality [1,82]. Thus, this finding highlights the need to use an appropriate measure of innovative human capital to estimate its role in economic and environmental quality for achieving economic and environment-related goals.

The results further indicate that the population density exerts a positive effect on carbon emissions. China ranks as one of the most populous countries globally, with a population estimated at 1.4 billion as of 2017. The population density was 138.5 per square kilometer in 2003 and 472 in 2017, which shows a 244% increase. Thus, greater population density stimulates human activities, which exert significant pressure on natural resources and the environment. Our findings are consistent with the results of Rahman and Alam [83] for Bangladesh and Ohlan [84] for India but against the results of Meng and Han [85].

The coefficients of structural change and energy intensity are 0.193% and 0.710%. These are statistically significant at a 1% level, indicating that if structural change and energy intensity increase (decrease) by 1%, then carbon emissions will increase (decrease) by 0.193% and 0.710%. The positive effect of structural change on carbon emission shows that structural change in China is not environmentally friendly. More precisely, the structural change deteriorates environmental quality. Energy intensity also damages environmental quality in China. The coefficient of FDI is negative and statistically significant. The negative effect of foreign direct investment on carbon emissions opposes the notion of the pollution haven hypothesis. These results support the view that foreign direct inflows promote environmental sustainability in China. It implies that China has strictly regulated its FDI and foreign investors do not transfer dirty technology to China.

Our findings of structural change are in line with the work of Ahmad et al. [79] but contrary to the results of Marsiglio et al. [86], and Ali et al. [87], who found that structural change improves environmental quality. The difference in results is because the structural change in China has increased industrialization that mainly relies on pollutant fossil fuels that degrade environmental quality. The outcome of energy intensity supports the previous work of Amin and Dogan [86] and Zhang and Zhou [87]. The result of FDI matches the outcome of Saud et al. [88] in the context of belt and road nations but contradicts the outcome of Shahbaz et al. [89], who found the deteriorating effect of FDI in France.

The outcomes of system GMM shown in Table 7 indicate that the results are consistent with the OLS and fixed effect estimates. The results depict that the coefficient for GDP is positive while the square of GDP is negative, which validates the EKC hypothesis in China. The innovative human capital has a negative and significant value, and the effects of structural change, population density, and energy intensity on carbon emissions are significant and positive. By employing the system GMM method the long-run coefficients of GDP, GDP square, innovation human capital, population density, structural change, energy intensity, and foreign direct investment are 0.543, −0.027, −0.027, 0.028, 0.174, 0.044, and −0.018, respectively.

## 6. Conclusions and Policy Suggestions

This paper inspected the role of innovative human capital (IHC) in CO_2_ emissions using a provincial dataset from 30 Chinese provinces from 2003 to 2017 and measuring innovative human capital based on the number of patents every one million R&D staff full-time equivalent. The results of fixed effect, OLS, and SYS-GMM methods revealed that innovative human capital decreases emissions in China and helps to form the EKC between CO_2_ emissions and GDP. In addition to this, economic structure, FDI, and energy intensity increase emissions, while population density lessens emission levels. The finding of the study reveals that China’s economic development will not be detrimental to environmental quality if IHC could be continuously developed.

Based on the above empirical results and conclusions, the paper proposes the following policies to realize carbon neutrality and to finally embark on the road of green development:

China needs to intensify efforts to foster innovative human capital. The paper’s empirical result shows that innovative human capital can bring a positive influence on reducing CO_2_ emissions. However, compared with the United States, Britain, Germany, and other Western developed countries, the quantity or quality of China’s innovative human capital is still at a comparatively low level. Therefore, from the national level, the Chinese central government needs to do some strategic planning to nurture innovative human capital. In this regard, launching different training programs for human capital, and focusing on increasing R&D staff by offering some subsidies and benefits on R&D in different sectors of the economy can help to foster innovative human capital. Moreover, policies should be designed to develop collaboration between universities and industries and research funding for universities should be increased to stimulate innovation. The collaboration of industries with the universities will enable them to reap the benefits of innovation and develop advanced technologies, which in turn will increase energy efficiency and reduce emissions.

The independent variable STRU denotes the proportion of annual industrial added value in GDP. The coefficient of STRU is positive indicating that it adds to emissions. Hence, the Chinese government should optimize the economic structure of China. There are two paths to optimize it. One is limiting or stopping the development of the heavy-polluting industry and promoting the application of environmentally friendly production technologies among all industries. The other is continually increasing the proportion of the modern service industry in the economic structure. The independent variable energy intensity increases emissions. The main problem is that the consumption percentage of fossil energy is still relatively high in China. The Chinese government must take strong measures to decrease the consumption of fossil energy, at the same time, efforts are needed to increase the use of renewable energy. Population density also drives emissions. China is the country that has the largest population in the world, and its average population density is also comparably high. Fortunately, in recent years, China’s population growth has been strictly controlled, and the growth rate has reduced. The Chinese government needs to initiate centralized city development to reduce the adverse effects of population density. Additionally, the focus should be on continuously improving public transportation and discouraging private vehicle ownership through different policies and taxes.

The coefficient of FDI in this study is negative. China is the largest developing country in the world. FDI is very important to China, especially when China has just implemented the policy of opening up. Through FDI, the Chinese government can make use of foreign investment to increase the research and development of green production technology, and this can also help to foster innovative human capital. Although China is now the second-largest economy in the world, its GDP per capita ranking is still relatively low. FDI is still very useful for China’s social and economic development, so China should keep collaborating well with developed countries and fully use its FDI to boost economic progress and environmental sustainability. In this regard, policies should be launched to ease foreign investment, and unnecessary barriers should be eliminated. The Chinese government should devise policies to direct FDI flow to the areas with relatively low innovative human capital. This will also promote the research and development of environmentally friendly production technology. Thus, China can embark on the road of green development and realize the strategic goals of reducing CO_2_ emissions as soon as possible.

This research explores a new topic and therefore, an extensive research gap exists that future studies can address. The innovative human capital based on R&D staff is directly linked with innovation and technological advancement. Thus, this measure is more appropriate to capture the impacts of IHC on the environment. However, this measure ignores the research output of university students (Master and Doctoral level). Therefore, future studies may use proxies including R&D staff and educational attainment for innovative human capital and compare the results of both methods. In addition, this paper used FDI inflows and ignored the potential reverse technology spillover. Future studies can expand the research on FDI in China and other countries by considering the reverse technology spillover effect. Researchers can examine the role of innovative human capital in economic growth for different nations using the new measure presented in this paper. Additionally, the impact of IHC on different environmental indicators can be investigated.

## Figures and Tables

**Table 1:** Summary of literature on human capital and environmental quality nexus.

Reference	Country	Period	Human Capital Proxy	Results
Danish et al. [17]	Pakistan	1971–2014	Human capital index (HCI)	HC mitigates ecological footprint
Yao et al. [19]	OEDC countries	1870–2014	Total number of average schooling years	HC has a favorable impact on the environment
Khan et al. [21]	122 countries	1980–2014	Primary, secondary, tertiary, and average years of schooling	HC improves the quality of the environment
Hao et al. [22]	G-7	1991–2017	Human capital index (HCI)	HC improves environmental quality
Mahmood et al. [27]	Pakistan	1980–2014	Human capital index (HCI)	HC mitigates emissions
Wang and Wu [7]	China and India	2013–2018	Stock of technological innovative professionals (10 thousand)	Improves air quality (reduces PM2.5)
Khan et al. [24]	OECD countries	1990–2018	Returns to education by incorporating information from the labor market	HC decreases emissions
Bano et al. [28]	Pakistan	1971–2014	Number of students enrolled in secondary school for general education per capita, number of students enrolled in secondary school for vocational education per capita, total number of students enrolled in secondary school as a percentage of gross enrollment, and human capital index (HCI)	HC reduces emissions
Huang et al. [31]	China	1998–2017	Heterogeneous human capital based on skilled and interprovincial migration rate.	HC decreases carbon intensity
Zafar et al. [32]	United States	1970–2015	Human capital index (HCI)	HC decreases pollution
Ahmed and Wang Ahmed et al. [33]	India	1971–2014	Human capital index (HCI)	HC improves environmental quality
Ahmed et al. [34]	G-7	1971–2014	Human capital index (HCI)	HC improves environmental quality
Ahmed et al. [35]	15 LCA countries	1995–2017	Human capital index (HCI)	HC increases emissions

Note. HC—human capital; LCA—Latin American and Caribbean countries; HCI refers to human capital index based on the average years of schooling and projected rate of return.

**Table 2:** Variable’s measurement, symbol, and data source.

Variables	Symbol	Measurement	Data Source
CO_2_ emissions	CO_2_	Total annual emissions based on different fossil fuel usage (measured in terms of metric tons)	CESY
Economic growth	GDP	GDP per capita (constant 2003 prices in RMB)	CPSY
Innovative human capital	IHC	The number of patents every one million R&D staff full-time equivalent.	CPSY and CSTY
Population density	POP	Population per square km of land	CPSY
Economic structure	STRU	The proportion of annual industrial added value in GDP	CPSY and CSTY
Energy intensity	ENER	Energy consumption (ton of standard coal equivalent) divided by GDP	CESY
Foreign direct investment	FDI	FDI’s inflows percentage of GDP	CPSY

Note: CESY—China energy statistical yearbook (https://data.cnki.net/Yearbook/ accessed on 16 June 2021); CPSY—Chinese provincial statistical yearbook (http://www.stats.gov.cn/tjsj/ndsj/ accessed on 16 June 2021); CSTY—China science and technology statistical yearbook (http://www.stats.gov.cn/ztjc/ztsj/kjndsj/ accessed on 16 June 2021); CESY—China environmental statistical yearbook (https://data.cnki.net/yearbook/Single/ accessed on 16 June 2021).

**Table 3:** Carbon emission factors.

Serial Number	Emission Source Name	Emission Factor
1	Gasoline	2.9251
2	Kerosene	3.0179
3	Diesel	3.0959
4	Fuel oil	3.1705
5	Raw coal	1.9003
6	Coke	2.8604
7	Natural gas	2.1622
8	Cement production	0.538

**Table 4:** Descriptive statistics.

Variable	Obs	Mean	Std. Dev.	Min	Max
CO_2_	450	261.529	181.965	15.600	842.200
GDP	450	25884.9	18029.369	3603	99783.0
IHC	450	2143.165	1402.827	264.25	6776.56
POP	450	436.756	633.98	7.000	3826.0
STRU	450	39.195	8.403	11.84	59.240
ENER	450	1.172	0.740	0.250	6.740
FDI	450	3.022	2.338	0.040	15.330

**Table 5:** Pairwise correlations.

	CO_2_	GDP	IHC	POP	STRU	ENER	FDI
CO_2_	1.000						
GDP	0.389 *	1.000					
GDP^2^	0.380 *	0.999 *	1.000				
IHC	0.369 *	0.651 *	0.645 *	1.000			
POP	0.285 *	0.435 *	0.445 *	0.320 *	1.000		
STRU	0.546 *	0.015	0.006	−0.089	0.009	1.000	
ENER	−0.261 *	−0.740 *	−0.739 *	−0.694 *	−0.573 *	0.162 *	1.000
FDI	0.018	0.198 *	0.204 *	0.072	0.569 *	0.037	−0.254 *

* *p* < 0.1.

**Table 6:** Regression results.

	OLS (1)		Fixed Effect (2)	
Variables	Coefficient	Std. Error	Coefficient	Std. Error
GDP	2.479 **	1.129	2.376 ***	0.359
GDP^2^	−0.115 **	0.057	−0.062 ***	0.019
IHC	−0.358 ***	0.055	−0.052 ***	0.018
POP	0.187 ***	0.047	0.257 **	0.110
STRU	0.049 ***	0.003	0.193 ***	0.053
ENER	0.200 **	0.094	0.710 ***	0.065
FDI	−0.178 ***	0.042	−0.034 **	0.014
Constant	−13.448 **	5.603	−12.414 ***	1.73
Observation	450	-	450	-
R^2^	0.525	-	0.788	-
Hausman test (Prob)	-	-	22.40 (0.002)	-
Province FE	-	-	Yes	-
Year FE	-	-	Yes	-

*** *p* < 0.01, ** *p* < 0.05.

**Table 7:** System GMM results.

Variables	Coefficient	Stand Error	T-Value	*p*-Value
L.CO_2_	0.896 ***	0.049	18.38	0.000
GDP	0.543 ***	0.131	4.14	0.000
GDP^2^	−0.027 ***	0.007	−4.12	0.000
IHC	−0.027 **	0.010	−2.62	0.014
POP	0.028 **	0.012	2.29	0.029
STRU	0.174 *	0.086	2.01	0.054
ENER	0.044 ***	0.015	2.87	0.008
FDI	−0.018 ***	0.005	−3.39	0.002
Constant	−3.307	7.590	−0.44	0.666
Sargan	121.76	-	-	-
P(Sargan)	0.100	-	-	-
AR(1)	0.534	-	-	-
AR(2)	0.013	-	-	-
Year fixed effect	Yes	-	-	-
ID fixed effect	Yes	-	-	-
Observations	415	-	-	-

Sargan test specifies the over-identification test for the restriction in system GMM estimation. The AR (1) and AR (2) Arellano–Bond test represents the first and second-order autocorrelation in the first difference. *** *p* < 0.01, ** *p* < 0.05, * *p* < 0.1.

## Data Availability

Data is readily available at request.
